# Comparison of T24H-his, GST-T24H and GST-Ts8B2 recombinant antigens in western blot, ELISA and multiplex bead-based assay for diagnosis of neurocysticercosis

**DOI:** 10.1186/s13071-017-2160-2

**Published:** 2017-05-15

**Authors:** Ana Hernández-González, John Noh, María Jesús Perteguer, Teresa Gárate, Sukwan Handali

**Affiliations:** 1 Helminth Unit, Parasitology Department, Centro Nacional de Microbiología, Instituto de Salud Carlos III , Crtra. Majadahonda-Pozuelo, km 2.2, 28220, Majadahonda, 28220 Madrid Spain; 2 Division of Parasitic Diseases and Malaria, Center for Global Health, Centers for Disease Control and Prevention, Atlanta, 30329 GA USA

**Keywords:** Neurocysticercosis, *Taenia solium*, Recombinant antigen, ELISA, Western blot, Multiplex bead-based assay

## Abstract

**Background:**

Currently, the reference standard assay for the serodiagnosis of neurocysticercosis (NCC) is the lentil lectin-bound glycoproteins/enzyme-linked immunoelectrotransfer blot (LLGP-EITB). The main disadvantage of this technique is the complexity of obtaining and purifying the LLGP extract. This could be solved by replacement with highly specific recombinant antigens from *Taenia solium*. Based on previous studies, we selected and produced the recombinant Ts8B2 and T24H proteins and applied them to three diagnostic techniques: western blot (WB), enzyme-linked immunosorbent assay (ELISA) and the multiplex bead-based assay (MBA).

**Methods:**

The Ts8B2 and T24H cDNA sequences were expressed in a prokaryotic system and the corresponding expression products purified; three recombinant proteins were further characterized: T24H-his, GST-T24H and GST-Ts8B2. The proteins on WB, ELISA and MBA were tested against 149 sera from patients with NCC confirmed by brain imaging, 40 sera from patients with other parasitic diseases, and 131 sera from US. individuals without evidence of neurocysticercosis (clinical/serological/brain imaging). The sensitivity and specificity of each antigen by WB were calculated by counting the number of true positive, false positive, true negative and false negative results. Using the receiver operating characteristic (ROC) curves, the cut-off values for the ELISA and MBA were established as well as the sensitivity and specificity of each assay.

**Results:**

All three antigens showed a high sensitivity on WB in active NCC cases with two or more viable cysts and low sensitivity for cases with single viable cyst or calcified lesions and inactive NCC. WB showed the highest specificity and sensitivity out of the three diagnostic techniques. The recombinant T24H-his was the best diagnostic reagent in WB (100% sensitivity, 99.4% specificity), exhibiting similar results to the LLGP-EITB, against the same panel of NCC sera. The GST-T24H antigen worked better than the others in ELISA and MBA protocols (88.3 and 96.1% sensitivity, respectively and 96.5% specificity).

**Conclusions:**

The sensitivity and specificity that we obtained were similar to results from a previous study using a similar recombinant antigen (rT24H), suggesting that recombinant antigens may be good alternatives to crude extracts in a variety of diagnostic techniques. Furthermore, these antigens can be applied in the development of point-of-care tests which would be useful in NCC field studies.

## Background

Human neurocysticercosis (NCC), caused by the larval stage of *Taenia solium*, is the most common helminth infection of the central nervous system and the most frequent cause of late-onset seizures in developing countries [1]. In Europe and the USA, reported cases are usually imported or related to contact with immigrant adult carriers of *T. solium*, but there are also a small number of autochthonous cases [2, 3]. Globally, the disease shows an annual incidence of 370,710 (95% uncertainty intervals, UI: 282,937–478,123), with 28,114 deaths (95% UI: 21,059–36,915) resulting 2,788,426 Disability-adjusted life years (DALYs) (95% UI: 2,137,613–3,606,582) lost [4].

NCC diagnosis is critical for deciding proper treatment as well as identifying sources of infection and hot spots, so that control strategies can be established [5, 6]. Because symptoms are nonspecific, the diagnosis must be based on a combination of the results obtained by imaging, serological tests and exposure history [7, 8]. Computed tomography (CT) and magnetic resonance imaging (MRI) are considered the reference techniques as imaging methods. Among serological tests, the reference standard is lentil lectin-bound purified glycoproteins (LLGP) in an enzyme-linked immunoelectrotransfer blot assay (EITB) [7, 9].

The LLGP-EITB test is a western blot (WB) assay for antibody detection to one or more of the seven specific glycoproteins from *T. solium* cysts. The described specificity is 100% and the sensitivity is dependent on factors such as the viability and the number of cysts; the test is able to recognize the 95% of cases with multiple viable cysts [9], while in cases with a single or calcified cyst, the sensitivity is reduced to 50–80% [10, 11]. The main drawbacks of this technique are the complex purification of the glycoprotein extract from cysts collected from naturally infected pigs and the difficulty in standardization [9]. Additionally, the WB is not an ideal technique for testing large numbers of samples. Furthermore, this purified extract includes non-specific parasitic proteins, and for this reason cannot be applied to other non-visual tests such as the enzyme-linked immunosorbent assay (ELISA) (as other platforms could not separate the specific from the non-specific parasite proteins).

Since the LLGP antigen is purified from native *T. solium* cysts with multiple target proteins, it is a major challenge to use in other diagnostic formats. An alternative to the LLGP antigen would be a highly specific recombinant antigen (s) from *T. solium*. Using recombinant proteins will not only be cheaper, but also cleaner, allowing it to be applied on different immunological tests such as the ELISA and/or multiplex, fluorescence bead-based assay (MBA) platforms. The multiplex bead-based assay can be used to determine the performance of different *Taenia* purified antigens in one assay and possibly improve the sensitivity of the assay. A multiplex platform could also give rise to a multi-parasite detection assay by combining different purified proteins from other parasites to detect other distinct parasitic diseases [12].

There is a long list of recombinant antigens and synthetic peptides that have been developed, using various techniques, for the serodiagnosis of NCC [13]. The recombinant GP50 and T24H [14, 15], derived from the 50 kDa and 24–42 kDa bands from the LLGP, have been tested in techniques such as multi antigen printing immunoassay (MAPIA), ELISA, western blot, QuickELISA and rapid Lateral Flow assay [15–18], with equally high specificity and sensitivity comparable to the reference standard [19].

Other recombinant antigens were characterized and cloned from the *T. solium* metacestode 8 kDa diagnostic antigen family [20, 21]. Ts8B1, Ts8B2 and Ts8B3 were assayed, as recombinant proteins, in an ELISA. Ts8B2 was shown to be the most sensitive and specific antigen.

Based on the sensitivity and specificity of these recombinant antigens, we selected the genes corresponding to Ts8B2 and T24H antigens to express them and evaluate their diagnostic properties in NCC immunodiagnosis. These antigens were obtained as recombinant proteins in a prokaryotic system and subsequently the sensitivity and specificity of these antigens were compared using three different test formats (WB, ELISA and MBA) for NCC detection.

## Methods

### Recombinant proteins production

#### GST-Ts8B2

The Ts8B2 sequence (GenBank: AJ508918.1) was subcloned into the expression vector pGEX-6P-2 (28-9546-50, GE Healthcare, Little Chalfont, UK) without the signal peptide, with a size of 198 bp. BL21 *Escherichia coli* competent cells (200133, Agilent Technologies, Santa Clara, CA, USA) were transformed with this construct. Induction of expression and subsequent purification, as a fusion protein to the Glutathione-S-Transferase (GST) tag, was performed as described in Ferrer et al. [20]. After dialysis against phosphate-buffered saline (PBS) pH 7.2, the protein GST-Ts8B2 was lyophilized and stored until use. Later, the powder was dissolved in water and the protein concentration was calculated by the Bradford method (Bio-Rad, Hercules, CA, USA).

#### GST-T24H AND T24H-his

The sequence corresponding to the hydrophilic extracellular domain of the T24 antigen (rT24H, GenBank: AY211879.1), described by Hancock et al. [15], was sub-cloned into the prokaryotic expression vector pGEX-6P-2 (28-9546-48, GE Healthcare). We added 18 nucleotides at 3′end corresponding to 6 histidines. Sub-cloning was performed by digestion with *EcoRI* and *NotI* restriction enzymes (Roche Farma, Madrid, Spain). The ligation products were used to transform the BL21 *E. coli* competent cells (Agilent Technologies, Santa Clara, CA, USA). Protein expression was induced with 0.5 mM isopropyl-β-D-thiogalactopyranoside (IPTG, I6758, Sigma-Aldrich, Saint Louis, MO, USA) at 16 °C and 220 rpm shaking overnight. The expected result after the expression is a 36 kDa recombinant protein with a GST tag at amino-terminal end and a polyhistidine tag at carboxy-terminal end. The protein purification from the bacterial lysate was carried out by affinity chromatography in two steps, with two different types of sepharoses: first we used a Glutathione Sepharose 4B (17-0756-01, GE Healthcare) and later a Ni Sepharose 6 Fast Flow (17-5318-01, GE Healthcare). We followed the purification protocol described by Corstjens et al. [16], with some modifications as we kept the polyhistidine tail. The T24H-his recombinant protein was eluted from the Glutathione Sepharose 4B column after PreScission Protease (27-0843-01, GE Healthcare) cleaved and in the case of the GST-T24H, after incubation with L-Glutathione reduced (G4251, Sigma-Aldrich). We performed a second purification on Ni Sepharose 6 Fast Flow column (as both recombinant proteins were still fused to the polyhistidine tag) and eluted with imidazole 500 mM (56750, Sigma-Aldrich, St. Louis, MO, USA). After dialysis, the GST-T24H and T24H-his recombinant proteins were lyophilized and stored until use. After resuspension in water, the proteins concentration was calculated by the colorimetric method of the Bradford (Bio-Rad, Hercules, CA, USA).

### Serum samples

For setting up the western blot, a pool of five sera from NCC patients (with positive results to the seven LLGP bands) was used as a positive control and a pool of five sera from negative (by clinical, serological and radiological examinations), non-traveler USA subjects as a negative control. In addition, serum from a patient with alveolar hydatid disease was used to determine if there was cross-reactivity with our assay. To set up the ELISA and multiplex fluorescence bead-based assay techniques, we used a pool of five sera from NCC patients with a medium level of antibodies as a positive control [optical density (OD) approximately equal to 1 for ELISA, or median fluorescence intensity (MFI) approximately equal to 10,000 for MBA] and the same pool of five sera from negative USA subjects as a negative control.

Once the working conditions were set up for each antigen and each test, three sets of defined sera were tested (Table 1). Set 1 (*n* = 149) included sera from NCC-defined serum samples obtained at the Instituto de Ciencias Neurologicas (Lima, Peru) and was used for determining sensitivity of the assay. The definitive diagnosis of NCC was confirmed by computer tomography or magnetic resonance imaging brain imaging. These samples were from patients categorized as having two or more viable cysts (*n* = 77), a single, viable cyst (*n* = 26) or calcified (non-viable) cysts only (*n* = 46). Of these 149 samples, 129 were seropositive for LLGP-EITB. Set 2 (*n* = 131) were obtained from residents of the USA who were determined to be negative for NCC based on clinical, serological and radiologic assessment. Set 3 (*n* = 40) were from patients with other infections based on parasitological and serological examinations. Set 2 and set 3 were used for establishing the specificity of the assay.

**Table 1:** Characteristics of patients with neurocysticercosis (NCC) (cyst viability, cyst number) and other subjects (subjects determined to be negative for NCC and subjects with other infections) whose serum samples were used to determine NCC recombinant antigen sensitivity and specificity by western blot, ELISA and MBA

Sera categories	Total number of sera
NCC cases (viable/non-viable) (Set 1)	
One viable cyst	26
Two or more viable cysts	77
Only calcified (non-viable) cysts	46
Total	149
Subjects negative for NCC (Set 2)	131
Subjects with other infections (Set 3)	
Hydatid echinococcosis	11
Alveolar echinococcosis	1
Strongyloidiasis	9
Schistosomiasis	9
Malaria	1
Toxoplasmosis	2
Trichinellosis	4
Filariosis	3
Total other parasites	40

### Western blot

All recombinant antigens tested (T24H-his, GST-T24H and GST-Ts8B2) in different concentrations (25, 12.5, 6.2, 3.1 ng/mm, etc., based on the amount of antigen used per mm of the strip length) were treated with 0.1% sodium dodecyl sulfate (SDS), in the absence of any reducing agents, by heating at 65 °C for 15 min [15] and then separated by molecular weight in 4–20% polyacrylamide gels (5671093, Bio-Rad). After electrophoresis, the proteins were transferred to nitrocellulose membrane (10-541-103, GE Healthcare).

First, each antigen concentration was assayed with the positive, negative and cross-reactor controls to select the most discriminatory antigen concentration to be used. The chosen antigen amount to evaluate the test was the concentration in which no signal was observed at the negative and cross-reactor sera, while the signal was kept against serum samples from individuals with NCC. Each antigen, at the selected concentration, was run on a 4–20% preparative polyacrylamide gel. After being transferred to nitrocellulose membrane, the blots were cut into 2.5 mm wide strips. GST-Ts8B2 and T24H-his recombinant proteins were run on the same gel and tested simultaneously on the same nitrocellulose strip; this was possible since they have different molecular weight and distinct epitopes for different antibodies. The strips were incubated against individual sera diluted 1:100 in PBS-0.3% Tween-20 and 5% dry skim milk (Table 1) for human antibody detection using in-house horseradish peroxidase conjugated goat antibodies to human IgG [14, 22]. The western blot was carried out following the protocol described in Tsang et al. [9, 23].

### ELISA

An ELISA test was optimized for each recombinant protein in ninety-six well Immulon 2HB plates (10111005, Thermo Scientific, Rockford, IL, USA) using the positive and negative controls. A checker-board titration was carried out by doing 2-fold serial dilution of antigen, serum and conjugate used. Different antigen concentrations (from 10 μg/ml to 0.01 μg/ml), different serum dilutions (from 1/50 to 1/400) and different conjugated antibody dilutions (from 1/1000 to 1/32,000) were tested. The development time for each recombinant antigen was established by a kinetic curve obtained by the readings made every 30 s for 15 min after addition of a peroxidase substrate (SureBlue tetra methylene blue (TMB) Microwell reagent Peroxidase Substrate-KPL, 52-00-00). The decision to choose the best condition depended on the ratio of the positive control signal to the negative control signal (signal-to-noise ratio).

The working conditions selected to further evaluate each recombinant protein with the 320 sera were as follow. Different plates were used for each antigen. Wells were sensitized with the antigens in 100 μl of coating buffer (50 mM Tris-HCl, pH 8, 2 mM EDTA, 1 M KCl) at 4 °C overnight. The plates were washed four times with PBS-Tween-20 (0.3%). Then 100 μl/well of serum diluted in PBS-0.3% Tween-20 and 5% skim dry milk was added. After incubating for 30 min on a plate shaker at room temperature, the plates were washed four times again. A mouse anti-human IgG labeled with horseradish peroxidase was used as a secondary antibody (9040–05, Southern Biotech, Birmingham, AL, USA). It was diluted in PBS-0.3%Tween-20 and 100 μl was added per well. The plates were incubated and washed as in the previous steps. After addition of 100 μl of SureBlue TMB Microwell reagent Peroxidase Substrate (52-00-00, KPL, Gaithersburg, MD, USA) and incubation on a plate shaker, the reaction was stopped with 100 μl of 1 N H_2_SO_4_. Finally, the plates were read at 450 nm using a VersaMax Kinetic ELISA Microplate Reader with SoftMax Pro v5.4 Software (Molecular Devices Corporation, Palo Alto, CA, USA) to determine the OD of each well.

### Multiplex, fluorescence bead-based assay

Recombinant proteins were coupled to the surface of the magnetic beads (MC10072-01 and MC10048-01 Bio-Plex Pro™ Magnetic COOH Beads, Bio-Rad) using 1-ethyl-3-[3-dimethylaminopropyl] carbodiimide hydrochloride (EDC, 341006 Calbiochem/EMD Millipore, Billerica, MA, USA) and N-hydroxysulfosuccinimide (Sulfo-NHS, 24510, Thermo Scientific), following the protocol described by Anderson et al. [12], except incubation of the antigen with the activated beads was carried out at 4 °C overnight under stirring.

Different protein concentrations were tested in the coupling protocol (from 50 μg/1.25*10E6 beads to 1 μg/1.25*10E6 beads). As with ELISA, the decision to choose the best condition depended on the signal-to-noise ratio. When the coupling with the antigen did not yield good results, a protocol described in Schlottmann et al. [24] using 4-(4,6- dimethoxy-(1,3,5) triazin-2-yl)-4-methyl-morpholinium chloride (DMTMM) reagent was tested. After identifying the best coupling conditions for each antigen, all the serum samples were tested. The GST-Ts8B2 and T24H-his antigens were coupled to a different code bead and assayed in the same well during the immunoassay.

The MagPlex immunoassays were performed on 96-well black round bottom plates (3792 Costar, Fisher Scientific) following the protocol described by Anderson et al. [12]. Briefly, sera were diluted at 1/100 in PBS-0.3% Tween-20 plus 5% skim milk and dispensed at 50 μl/well plus 2500 coupled beads diluted in 50 μl of the same buffer. After incubating for 30 min at room temperature on a plate shaker, the plates were washed twice using a Biotek Magnetic Washer EL × 50. Then, 50 μl of a conjugate biotinylated anti-Human IgG (9042–08, Southern Biotech) was applied to each well in a 1/200 dilution in PBS-1% bovine serum albumin (BSA). The plates were incubated and washed as indicated before. Subsequently, 50 μl of R-phycoerythrin-labeled Streptavidin conjugate (Invitrogen, S866, Carlsbad, CA, USA) in a 1/250 dilution in PBS-1% BSA were added. After incubation and washing, the beads were suspended in 100 μl of PBS-1% BSA. MFI from each well was determined by using the BioPlex manager software, version 6.02 (Bio-Rad) and a Luminex 100 platform.

### Statistical analysis

The sensitivity and specificity for each antigen in WB were calculated using the following formulas: sensitivity as TP/(TP + FN) and specificity as (TN/TN + FP), where TP is true positive, TN is true negative, FP is false positive and FN is false negative. Significant differences in sensitivity and specificity between antigens and serum groups were estimated by the Chi-square (*χ* ^2^) test for *P*-value < 0.001.

The data obtained from ELISA (OD) and MBA (MFI) for each sample were transformed into a serological index, calculated based on these formulas: for ELISA, the serological index = (OD _sample_ – OD _negative control_)/(OD positive control – OD _negative control_) and for the multiplex bead-based assay, the serological index = (MFI _sample_ – MFI _negative control_)/(MFI _positive control_ – MFI _negative control_). The cut-off values, sensitivity and specificity were established by receiver operating characteristics (ROC) curves using the serological index values for each antigen and test. The area under the ROC curve allowed us to compare the diagnostic performance between tests. Significant differences between tests were estimated by the Chi-square test for *P*-value < 0.05. All statistical analyzes were performed using SPSS version 22.

## Results

### Recombinant protein production

#### GST-Ts8B2

**Fig. 1:**
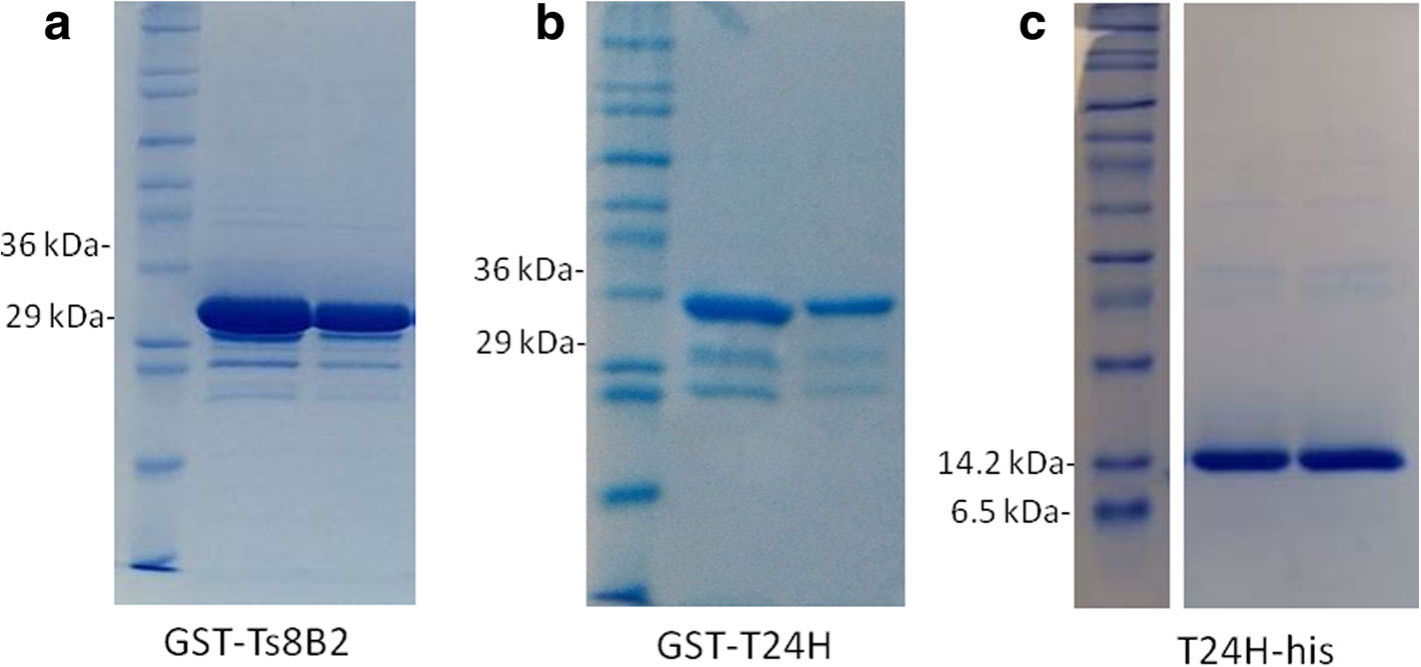
Electrophoresis in 12.5% SDS-PAGE of purified recombinant proteins. **a** GST-Ts8B2 (MW 33.6 kDa) after purification and dialysis, dyed with Coomassie Brilliant blue. **b** GST-T24H (MW 35.8 kDa) after purification and dialysis, dyed with Coomassie Brilliant blue. **c** T24H-his (MW 9.8 kDa) after purification and dialysis, dyed with Coomassie Brilliant blue

The GST-Ts8B2 fusion protein was expressed and purified with a molecular weight according to the expected size of 33.6 kDa (7.6 kDa from the Ts8B2 sequence without signal peptide plus 26 kDa from GST, Fig. 1a). The protein was obtained at 0.55 mg/ml, calculated by the Bradford colorimetric method.

#### GST-T24H and T24H-his

The GST-T24H fusion protein was expressed and purified with a molecular weight according to the expected size of 35.8 kDa (9.8 kDa from the T24H-his sequence without signal peptide plus 26 kDa from GST and the polyhistidine tag, Fig. 1b). The protein was obtained at 0.275 mg/ml, calculated by the Bradford colorimetric method. The T24H-his recombinant protein was obtained at 0.250 mg/ml with an approximate molecular weight of 10 kDa.

### Western blot

**Fig. 2:**
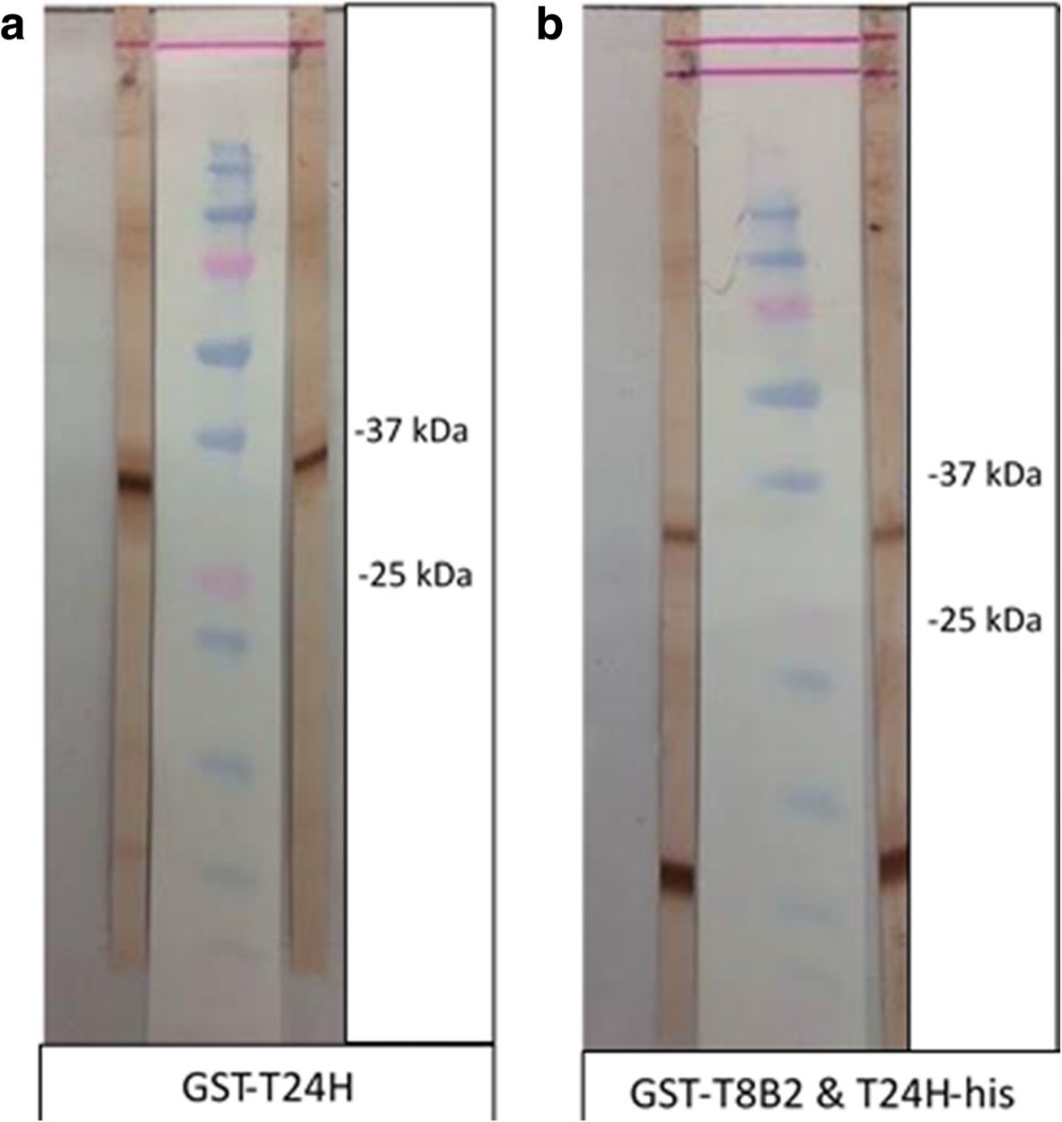
Recombinant proteins on the nitrocellulose membrane after western blot using neurocysticercosis (NCC)-positive control serum samples. **a** GST-T24H recombinant antigen (MW 35.8 kDa). **b** GST-Ts8B2 (MW 33.6 kDa) and T24H-his (MW 9.8 kDa) recombinant antigens on the same strip

The final concentrations for each antigen were the following: GST-T24H 1.5 ng/mm, GST-Ts8B2 1 ng/mm, and T24H-his 3 ng/mm (Fig. 2). The T24H-his showed the best signal-to-noise ratio and allowed the use of a higher antigen concentration that improved sensitivity without decreasing specificity. With the optimized antigen concentration in western blot format, T24H-his was the most sensitive antigen, independent of cyst category (calcified or viable) (Table 2), showing a 100% sensitivity for patients with 2 or more viable cysts.

**Table 2:** Sensitivity (S)^a^ and specificity (Sp) of results obtained in Western blot with T24H-his, GST-Ts8B2, GST-T24H, compared to LLGP-EITB test using the collection of serum samples described

			Sensitivity					
	Specificity (*n* = 171)		One viable cyst (*n* = 26)		Two or more viable cysts (*n* = 77)		Calcified cysts (*n* = 46)	
	(+/−)	Sp (%)	(+/−)	S (%)	(+/−)	S (%)	(+/−)	S (%)
T24H-his	1/170	99.4	23/3	88.5	77/0	100	38/8	82.6
GST-Ts8B2	2/169	98.8	19/7	73.1	68/9	88.3	16/30	34.8
GST-T24H	0/171	100	21/5	80.8	74/3	96.1	37/9	80.4
LLGP			22/4	84.6	75/2	97.4	32/11^b^	74.4

A Chi-square test (*χ* ^2^) carried out for each antigen, with degrees of freedom (*df*) equal to 1 and significance level (*P*) *P* < 0.05. T24H-his: *χ* ^2^ = 274.488, *df* = 1, *P* < 0.001; GST-Ts8B2: *χ* ^2^ = 169.289, *df* = 1, *P* < 0.001; GST-T24H, *χ* ^2^ = 257.855, *df* = 1, *P* < 0.001

*Abbreviations*: *n* number of sera in each group, *+/−* number of positive and negative sera obtained in each case, *Sp (%)* specificity percentage obtained with each antigen in western blot, *S (%)* sensitivity percentage for each antigen in each NCC group

^a^Sensitivity was determined according to viability and number of cysticerci in neurocysticercosis (NCC) patients

^b^Just 43 samples from patients with calcified cysts were tested in LLGP-EITB

The GST-T24H antigen showed 100% specificity and a lower sensitivity than the T24H-his recombinant protein in this technique. T24H-his showed a false positive result, weak intensity band recognition, with one negative USA individual. The GST-Ts8B2 antigen was the least sensitive and specific antigen, as two sera from healthy individuals reacted weakly. None of the recombinant antigens reacted with sera from patients with other parasitic infections (see Table 2). A Chi-square test was carried out for each antigen, with degrees of freedom (*df*) equal to 1 and significance level *P* < 0.05 (T24H-his: *χ* ^2^ = 274.488, *df* = 1, *P* < 0.001; GST-Ts8B2 *χ* ^2^ = *169.289*, *df* = 1, *P* < 0.001; GST-T24H: *χ* ^2^ = 257.855, *df* = 1, *P* < 0.001).

### ELISA

The best conditions for the antigens GST-T24H and T24H-his were obtained when they were applied at 0.325 μg/ml, when the sera dilution was made at 1/100 in PBS with 0.3% Tween-20 and 5% skim dry milk and when the secondary antibody was applied at 1/2000 in PBS-0.3%Tween-20. The best time to stop the reaction was in 3 min for GST-T24H and in 7.30 min for T24H-his.

The GST-Ts8B2 recombinant protein showed the best signal-to-noise ratio at 0.625 μg/ml concentration, at 1/100 sera dilution and at 1/4000 dilution for the secondary antibody. The reaction was carried out in 1.30 min.

**Fig. 3:**
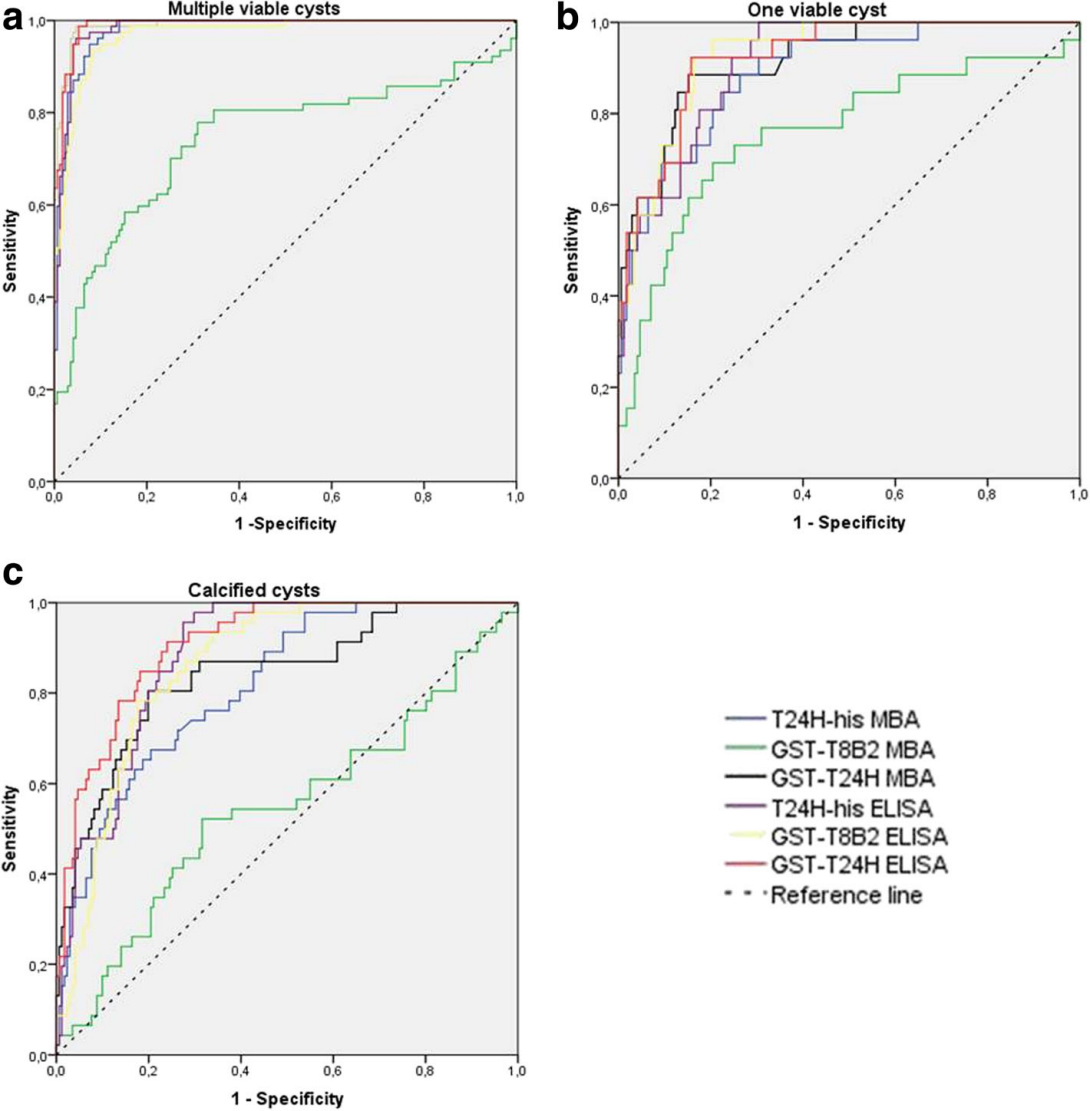
ROC curves of the different antigens tested in ELISA and MBA with serum samples from neurocysticercosis patients with two or more viable cysts (**a**), only one viable cyst (**b**) and only calcified (non-viable) cysts (**c**)

The cut-off value chosen for each test led to specificity above 95%. Under these conditions, and a fixed specificity at 96.5% by ROC curves, GST-T24H and T24H-his antigens showed similar sensitivity (88.3%) for patients with two or more viable cysts, whereas the GST-T24H seemed to detect a higher number of positive than T24H-his (53.8 *vs* 50%) with samples with one viable cyst; 43.5 *vs* 34.8% with samples with calcified cysts (Tables 3, 4 and 5; Fig. 3a–c). The GST-Ts8B2 showed the lowest sensitivity for all NCC cases in comparison to the other two antigens. Regarding specificity, the three recombinant antigens showed positive values for some sera in this technique: one serum from *E. granulosus* infection, and five from NCC-negative individuals in case of T24H-his, one serum from *E. granulosus* infection, one from *Plasmodium falciparum* infection and four from NCC-negative individuals in case of GST-T24H and six sera from NCC-negative individuals for the recombinant GST-Ts8B2.

**Table 3:** ELISA and MBA results for each antigen in neurocysticercosis patients with two or more viable cysts (*n* = 77)

Test	Area	95% CI	SE	*P*	Cut-off	S (%)	Sp (%)
T24H-his ELISA	0.98	0.971–0.996	0.006	< 0.001	23.3	88.3	96.5
GST-Ts8B2 ELISA	0.97	0.951–0.989	0.010	< 0.001	34.5	75.3	96.5
GST-T24H ELISA	0.99	0.982–0.998	0.004	< 0.001	21.1	88.3	96.5
T24H-his MBA	0.98	0.966–0.994	0.007	< 0.001	9.2	84.4	96.5
GST-Ts8B2 MBA	0.74	0.667–0.819	0.039	< 0.001	250.0	46.8	91.2
GST-T24H MBA	0.99	0.981–0.999	0.005	< 0.001	4.0	96.1	96.5

*Abbreviations*: *Area* area under the ROC curve, *CI* confidence interval, *SE* standard error, *Cut-off* serological index value selected as the cut-off for each test, *S (%)* sensitivity percentage, *Sp (%)* specificity percentage

**Table 4:** ELISA and MBA results for each antigen in neurocysticercosis patients with only one viable cyst (*n* = 26)

Just one viable cyst (*n* = 26)							
Test	Area	95% CI	SE	*P*	Cut-off	S (%)	SP (%)
T24H-his ELISA	0.91	0.863–0.956	0.024	< 0.001	23.3	50.0	96.5
GST-Ts8B2 ELISA	0.93	0.886–0.970	0.021	< 0.001	34.5	53.8	96.5
GST-T24H ELISA	0.92	0.878–0.971	0.024	< 0.001	21.1	53.8	96.5
T24H-his MBA	0.89	0.826–0.953	0.032	< 0.001	9.2	50.0	96.5
GST-Ts8B2 MBA	0.76	0.644–0.875	0.059	< 0.001	250.0	42.3	91.2
GST-T24H MBA	0.92	0.862–0.970	0.028	< 0.001	4.0	57.7	96.5

*Abbreviations*: *Area* area under the ROC curve, *CI* confidence interval, *SE* standard error, *Cut-off* serological index value selected as the cut-off for each test, *S (%)* sensitivity percentage, *Sp (%)* specificity percentage

**Table 5:** ELISA and MBA results for each antigen in neurocysticercosis patients with only calcified (non-viable) cysts (*n* = 46)

Calcified Cysts (*n* = 46)							
Test	Area	95% CI	SE	*P*	Cut-off	S (%)	SP (%)
T24H-his ELISA	0.88	0.841–0.928	0.022	< 0.001	23.3	34.8	96.5
GST-Ts8B2 ELISA	0.86	0.811–0.911	0.026	< 0.001	34.5	15.2	96.5
GST-T24H ELISA	0.91	0.869–-0.950	0.021	< 0.001	21.1	43.5	96.5
T24H-his MBA	0.82	0.754–0.881	0.032	< 0.001	9.2	32.6	96.5
GST-Ts8B2 MBA	0.55	0.445–0.647	0.052	0.341	250.0	13.0	91.2
GST-T24H MBA	0.84	0.774–0.909	0.034	< 0.001	4.0	37.0	96.5

*Abbreviations*: *Area* area under the ROC curve, *CI* confidence interval, *SE* standard error, *Cut-off* serological index value selected as the cut-off for each test, *S (%)* sensitivity percentage, *Sp (%)* specificity percentage

### Multiplex bead based assay

After determining different antigen concentrations to be coupled on the bead, 1 μg/scale of the GST-T24H and 12.5 μg/scale of T24H-his was used for further experiments. Different coupling protocols were tested with GST-Ts8B2 recombinant antigen [12, 24] but none worked optimally, as little differences between the positive and negative controls were observed. Finally, GST-Ts8B2 was used at 50 μg/scale, following the standard coupling protocol, to compare the performance with the other antigens in MBA.

The cut-off value chosen for each test led to specificity above 95%. The best results were achieved with the GST-T24H protein, with a sensitivity of 96.1% for the diagnosis of patients with two or more viable cysts. This recombinant protein remained the most sensitive antigen for other categories of NCC (i.e. only one viable cyst and only calcified non-viable cysts) compared to T24H-his and GST-Ts8B2. GST-T24H showed some cross-reactions to: one serum from *S. mansoni* infection, one from *E. granulosus* infection, and three from NCC-negative individuals. The three recombinant antigens were more sensitive in detecting antibodies in sera from NCC patients corresponding to multiple viable cysticerci. In addition, the GST-T24H showed a higher sensitivity in the MBA technique than in the ELISA test except for calcified cysts, whereas the T24H-his and GST-Ts8B2 antigens had higher or equivalent sensitivities in the ELISA test. The results obtained with each antigen in the MBA test are shown in Tables 3, 4, 5 and Fig. 3.

## Discussion

The objectives of this study were to obtain highly specific and sensitive recombinant antigens and to evaluate their application to various techniques for NCC serodiagnosis, in order to: (i) replace the use of *T. solium* crude extracts, and (ii) have different tests available according to the diagnostic needs; for instance, techniques to analyze a large number of samples (epidemiological work), assays to discriminate the different NCC forms, tests to be developed using new materials or with innovative methodologies techniques easy to handle in endemic regions, etc.

In this study we worked with various recombinant antigens corresponding to two genes, T24H and Ts8B2. T24H is the large hydrophilic, extracellular domain of 92 amino acids from the T24 integral membrane protein, which corresponds to the 24 and 42 kDa bands in the *T. solium*-LLGP diagnostic antigen [15]; it showed excellent diagnostic properties [15], although 73% of the T24H sequence is shared with two EST sequences from *Echinococcus granulosus* (BI244014 and BF643023). The Ts8B2 gene belongs to the 8 KDa antigen family, which is also a member of the LLGP extract [20]. In the past, we carried out the characterization of three diagnostic antigens belonging to the 8 kDa antigenic family, obtained by screening of an expression library of *T. solium* metacestode. The Ts8B2, an excretion/secretion protein, showed the best diagnostic performance with sera from NCC patients (sensitivity 96.8%, specificity 93.1%) [20]; it has a homology of 64% with the antigen B 8 kDa subunits of *E. granulosus*. Regarding the molecular similarities of the two antigens in both cestodes and the potential cross-reactivity, some authors postulated the existence of conformational epitopes very different between *E. granulosus* and *T. solium*, in spite of structural analogies [15], as well as different exposure to the immune system in the two parasitic cysts, according to their distinct structure or changes in the antigen release in different metacestode-stages [25]. However, the appearance of cross-reactions should not be ruled out. Based on these previous findings, we selected the sequences of the antigens Ts8B2 and T24H in order to produce them as recombinant proteins and evaluate their diagnosis efficacy in different immunodiagnostic formats.

The antigens were expressed in a prokaryotic system (*E. coli*) because this option was simpler and faster than the eukaryotic ones, and it is easy to accomplish a good yield. In this regard, Ferrer et al. [21] demonstrated similar diagnosis performance of the GST-Ts8B2 recombinant protein produced in both systems, prokaryotic (Plasmid-*E. coli*) and eukaryotic (Baculovirus-insect cells) for NCC diagnosis. In most of the previous studies comparing different antigens and techniques, T24H was expressed in a eukaryotic system [15, 17, 26]. However, our results show that T24H produced in *E. coli* has reactivity comparable to that obtained with the same protein expressed in baculovirus, with similar results to those described by Noh et al. [19] in Western blot using sera from patients with multiple active cysts. Corstjens et al. [16] expressed the recombinant T24H with GST tag in a prokaryotic vector and also showed good results compared with the recombinant antigen expressed in a eukaryotic system. The only difference between their protein and the T24H-his of the present paper is that they removed the poly-histidine tail at the carboxy-terminal end.

With respect to the effect of the various constructions, it is interesting to point out that GST-T24H and T24H-his, despite being the same antigen, showed different diagnostic properties; this suggests that the GST tail changed the antigenic characteristics of the molecule, modifying the diagnostic performances when the recombinant proteins were applied in distinct techniques. Thus, regarding the MBA, the coupling to magnetic beads gave better results when using the T24H fused to the GST tag, showing higher intensity values with sera from NCC patients and maintaining the same specificity than the protein without the GST tag. In this case, the GST tag could have caused the antigen epitopes that are recognized by the antibodies to be more exposed after the coupling to the beads and therefore the GST tag would have increased the sensitivity of the T24H molecule.

In the case of ELISA, T24H-his and GST-T24H showed equal sensitivity to serum samples from patients with two or more viable cysts, but when evaluating sera from NCC patients with only calcified cysts, GST-T24H showed a slightly higher sensitivity; perhaps this is related to the different structure of the two antigens, as described above.

Regarding WB, the protein T24H-his was the cleanest and most sensitive reagent and by this reasoning it was possible to apply it in a higher concentration than the rest of the tested proteins. In WB, T24H-his had one weak false positive, which was not detected with the protein containing the GST tag. We think that the test could achieve a specificity of 100% if the T24H-his concentration is decreased to 2.5 ng/mm, thus following the same protocol as described by Noh et al. [19], with the assumption of a possible loss in sensitivity.

For all immunological techniques tested, GST-Ts8B2 was the least reactive antigen. However, previous studies described higher sensitivity and specificity for this recombinant protein [20, 21]. These variations could be due to the different serum sample collections used in each case for the analysis of the diagnostic properties; differences in antigenic properties of NCC diagnostic molecules have been described with distinct serum collections from different geographic origins (27). In WB, this antigen was applied in a lower concentration than the rest of the antigens in order to avoid cross-reactions, achieving a good sensitivity of 88.3%, although in its evaluation we obtained two false positive results with sera from negative individuals. GST-Ts8B2 proved to be unsuitable in the MBA technique since the coupling to beads affected the reactivity of the protein so that the differences between positive and negative were not as marked as they were with the remaining antigens. GST-Ts8B2 antigen in ELISA showed acceptable results of sensitivity and specificity, although below the values obtained with T24H derivatives.

The three antigens in ELISA and MBA were recognized by one serum sample from a patient with hydatidosis and by some sera from negative individuals. Regarding the false positive with a sample corresponding to the hydatidosis case, it could be explained by the similarity between the T24H deduced amino acid sequence and some tetraspanins from *Echinococcus* spp. (around 63% identity) and the 64% identity between Ts8B2 amino acid sequence and the antigen B in *E. granulosus*, mentioned above. In addition, GST-T24H in MBA reacted with a serum from a patient infected with *S. mansoni*; although identities in the homologous sequences were not found; in other studies, the same antigen reacted with samples from patients with *S. mansoni* and eventually with some healthy donor serum [16, 17, 26]. Also, GST-Ts8B2 showed the lowest specificity, testing positive with a sample from a patient with *Plasmodium* spp. by MBA. This antigen has not been tested against *Plasmodium* spp. before and similarities have not been found between the two parasites; therefore, we assume that this false positive could be due to the problems found with the use of GST-Ts8B2 in MBA.

## Conclusions

The recombinant proteins GST-T24H and T24H-his are good alternatives to the LLGP antigen for the diagnosis of NCC, since the results were very similar for the recombinant molecules and the reference purified antigens in the same techniques, WB or EITB. Also, the recombinant proteins can be used with various immunodiagnostic techniques and this is an important advantage. After comparing the three antigens in three different techniques, WB with the T24H-his recombinant antigen showed the highest sensitivity and specificity with the serum collection used. Consequently, this would be the most suitable system for application in NCC diagnosis as a confirmatory test. Besides, its characteristics (specificity, sensitivity, easy production) suggest that the antigen could be applied to a point-of-care (POC) test to be employed in endemic regions [16, 26]. The ELISA and the MBA would be the techniques of choice for screening, with the advantage that the antigens in the MBA would enable the diagnosis of multiple parasitoses in a single assay with high sensitivity.

## Abbreviations

BSA: Bovine serum albumin
CT: Computerized tomography
DMTMM: 4-(4,6- dimethoxy-(1,3,5) triazin-2-yl)-4-methyl-morpholinium chloride
EDC: 1-ethyl-3-[3-dimethylaminopropyl] carbodiimide hydrochloride
ELISA: Enzyme-linked immunosorbent assay
GST: Glutathione-S-Transferase
IPTG: Isopropyl-β-D-thiogalactopyranoside
LLGP-EITB: Lentil lectin-bound glycoproteins/enzyme-linked immunoelectrotransfer blot
MAPIA: Multi-antigen printing immunoassay
MBA: Multiplex bead-based assay
MFI: Median fluorescence intensity
MRI: Magnetic resonance imaging
NCC: Neurocysticercosis
OD: Optical density
PBS: Phosphate buffered saline
ROC: Receiver operating characteristic
rpm: Rotation per minute
SDS: Sodium dodecyl sulfate
Sulfo-NHS: N-hydroxysulfosuccinimide
TMB: Tetra methylene blue
UI: Uncertainty intervals
WB: Western blot
